# Posterior reversible encephalopathy syndrome: role of transorbital ultrasound

**DOI:** 10.1007/s10072-020-04719-5

**Published:** 2020-09-22

**Authors:** Piergiorgio Lochner, Martin Lesmeister, Raffaele Nardone, Andrea Orioli, Antonio Siniscalchi, Andrea Naldi

**Affiliations:** 1 Department of Neurology, Saarland University Medical Center, Homburg, Germany; 2 Department of Neurology, Franz Tappeiner Hospital, Merano, Italy; 3 Department of Neurology, Christian Doppler Clinic, Paracelsus Medical University, Salzburg, Austria; 4 Department of Neurology and Stroke Unit, Annunziata Hospital, Cosenza, Italy; 5 Department of Neuroscience Rita Levi Montalcini, University of Turin, Turin, Italy

Dear Editor,

Posterior reversible encephalopathy syndrome (PRES) presents most often with unspecific symptoms such as headache, confusion, decreased alertness, visual dysfunction, and seizures, which sometimes develop to status epilepticus. The diagnosis should be suspected when some triggers (abrupt arterial hypertension, preeclampsia, immunosuppressive therapy, transplantation, autoimmune diseases, etc.) are associated with an encephalopathy syndrome in the context of typical magnetic resonance (MR) findings indicative of vasogenic edema (areas of increased signal on fluid-attenuated inversion recovery (FLAIR) predominantly localized to the posterior cerebral hemispheres) [1]. Although its pathogenesis is not completely understood, two hypotheses have been postulated: the first theory consists of a rapidly developing hypertension exceeding the upper limit of cerebral blood flow autoregulation and leading to increased cerebral perfusion, breakdown of the blood-brain barrier with endothelial dysfunction, and consequent extravasation of fluid and blood products into the brain parenchyma. The second theory supports endothelial damage due to direct effects of excessive circulation cytokines, promoted by immunosuppressive or cytotoxic drugs, or autoimmune disorders releasing vasoactive factors, which induce an increase of vascular permeability and interstitial brain edema [1].

In about 90% of patients, complete remission of clinical symptoms occurs within weeks and a restitution of abnormal conditions in MR images is observed in about 70% of cases, whereas complications are described in up to 10%, henceforth leading to death in 3–6% [2]. Therefore, rapid and early identification and strict monitoring of patients threatened with the worst outcome is needed. Because symptoms’ severity mostly depends on the development of high intracranial pressure (ICP), a bedside and quick tool for its detection may be particularly useful. Transorbital sonography (TOS) for optic nerve sheath diameter (ONSD) and optic disc elevation (ODE) assessment is a promising technique, which is able to identify raised ICP and may represent a valid alternative to the standard referring invasive methods for ICP measurement. However, to date, only one report about its application in patients with PRES is available, leaving unclear the role of this technique for monitoring PRES [3]. Therefore, we present a detailed description of a PRES case series in which repeated TOS examinations played a crucial role in the diagnostic phase and monitoring the course of the disease.

We performed TOS both acutely and over time in four consecutive patients with PRES admitted to the Department of Neurology at our hospital in 2018 and 2019. Clinical and demographic characteristics, neuroimaging aspects, diagnostic procedures, and ocular sonography findings are thoroughly presented in the table (Table 1). Based upon clinical features and typical MRI findings the diagnosis of PRES was made, subsequently ruling out other conditions such as encephalitis, malignancy, vasculitis, and leukoencephalopathy. All patients underwent CSF analysis, whereas CSF pressure was measured in two of them. A hypertensive state was documented in all cases. Two patients recovered completely, one only improved, and another one died of massive cerebral edema on the second day. MR and CT images of the deceased patient are shown in Fig. 1. The most significant vasogenic edema is depicted in Fig. 2. TOS was executed using a Vivid Seven ultrasound system (linear array probe 3.5–10 MHz; GE, Milwaukee, WI, USA) with the patients lying in supine position at 30° and the probe placed on the temporal region of the upper eyelid. ONSD was measured 3 mm behind the globe, defined as the distance between the hyperechogenic borders of the subarachnoidal space, while ODE was gauged between the fundus and the dome of the papilla (Fig. 3). Three ONSD measurements were repeated in the axial plane and their mean was used as the average value for each eye. TOS was executed prior to lumbar puncture, except for patient number four.

**Table 1:** Demographic and clinical profile, diagnostic procedures, neuroimaging aspects, and ultrasonography findings in four patients with PRES

Patient ID, age (years), sex	Days from symptoms onset	MAP (mmHg)	CSF pressure (mmHg)	MRI findings (bilateral regions involved)	Clinical features	ONSD (mm)		ODE (mm)	
						R	L	R	L
1, 54, F	4	130	41	Fronto-parieto-occipital lobes	Headache, visual loss, neuropsychiatric symptoms	6.3 ± 0.1	6.0 ± 0.1	1.4	1.6
	40	83	-	-	No symptoms	4.8 ± 0.2	4.9 ± 0.1	-	-
2, 39, M	5	110	30	Parieto-occipital lobes, thalamus	Headache, focal signs,neuropsychiatric symptoms	6.2 ± 0.1	5.9 ± 0.1	1.1	0.7
	55	85	-	-	No symptoms	5.4 ± 0.3	5.6 ± 0.1	-	-
3, 60, F	6	117	nd	Parieto-occipital lobes, cerebellum, thalamus	Neuropsychiatric symptoms, ataxia	6.7 ± 0.1	7 ± 0.1	-	-
	19	89	-	-	Mild ataxia	5.6 ± 0.2	5.6 ± 0.1	-	-
4, 66, F	1	115	nd	Fronto-parieto-occipital lobes, brainstem, thalamus	Headache, dizziness, focal signs, seizures and status epilepticus	6.2 ± 0.2	6.3 ± 0.1	-	-
	2	93	-	-	Dead	6.0 ± 0.1	6.0 ± 0.1	-	-

*ONSD*, optic nerve sheath diameter (values are expressed as mean ± standard deviation); *ODE*, optic disc elevation; *MAP*, mean arterial pressure; *CSF*, cerebrospinal fluid; *MR*, magnetic resonance; *R*, right; *L*, left; *M*, male; *F*, female; *ND*, not determined

**Fig. 1:**
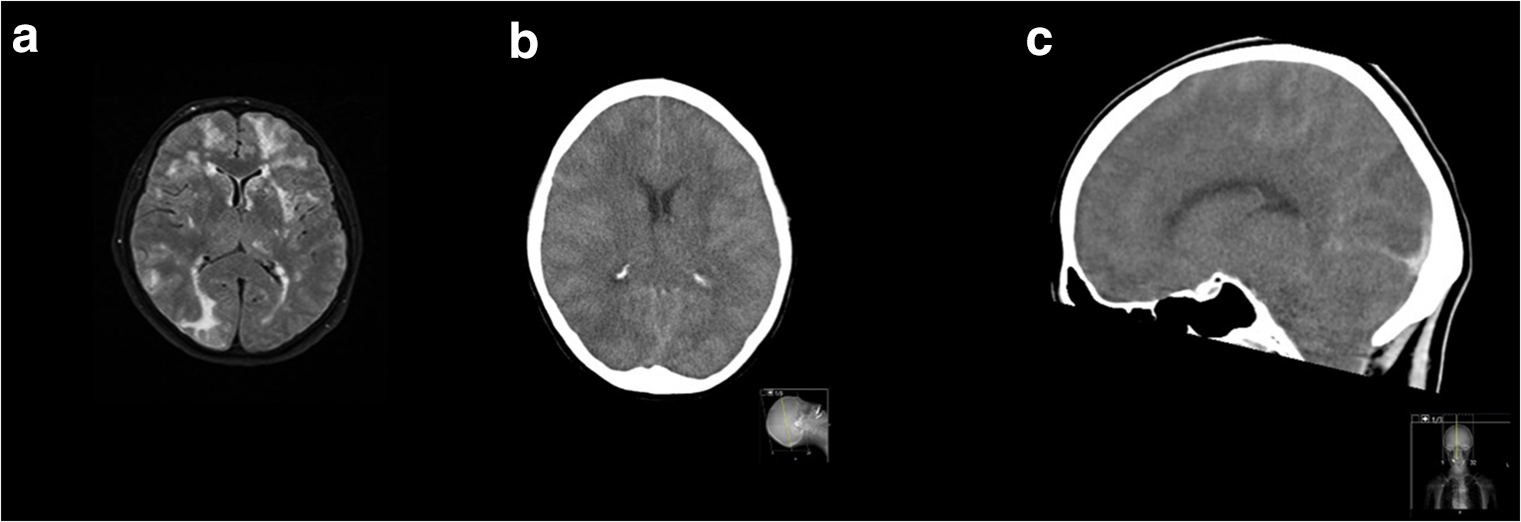
MR and CT imaging of patient 4. MR-T2* image showing diffuse low signal area due to blood products and hyperintense alteration involving predominantly and asymmetrically the frontal, insular and parieto-occipital regions bilaterally, compatible with vasogenic edema (**a**). CT brain showing diffuse effacement of sulci, consisting with cerebral edema (**b**, axial scan; **c**, sagittal view)

**Fig. 2:**
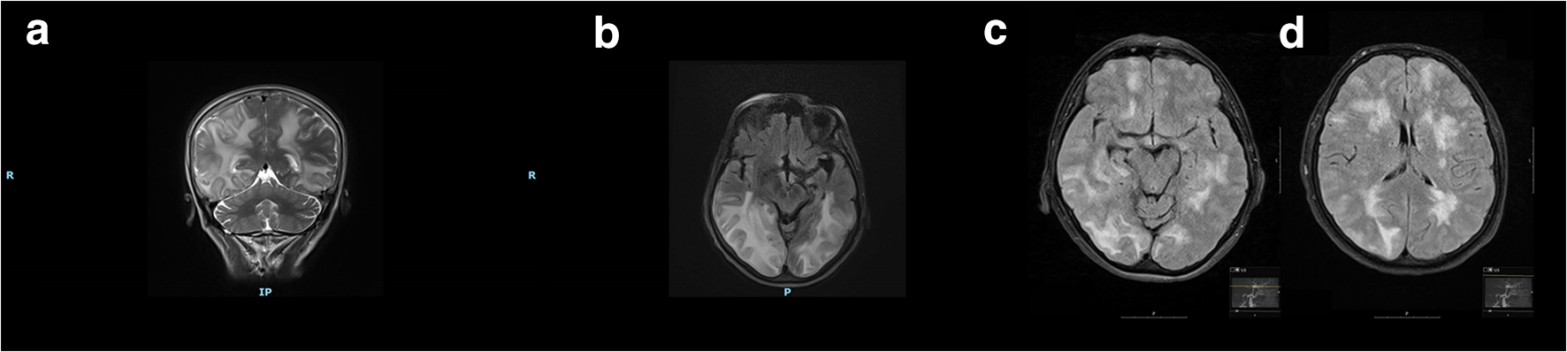
MR imaging of patient 1 with coronal T2 (**a**) and axial FLAIR (**b**) images showing the diffuse and symmetrical distribution of vasogenic edema in the cortical and subcortical area of fronto-parieto-occipital lobes. MR imaging of patient 4 with axial FLAIR (**c**, **d**) images showing hyperintensity of white matter representing vasogenic edema in the fronto-temporo-parieto-occipital area

**Fig. 3:**
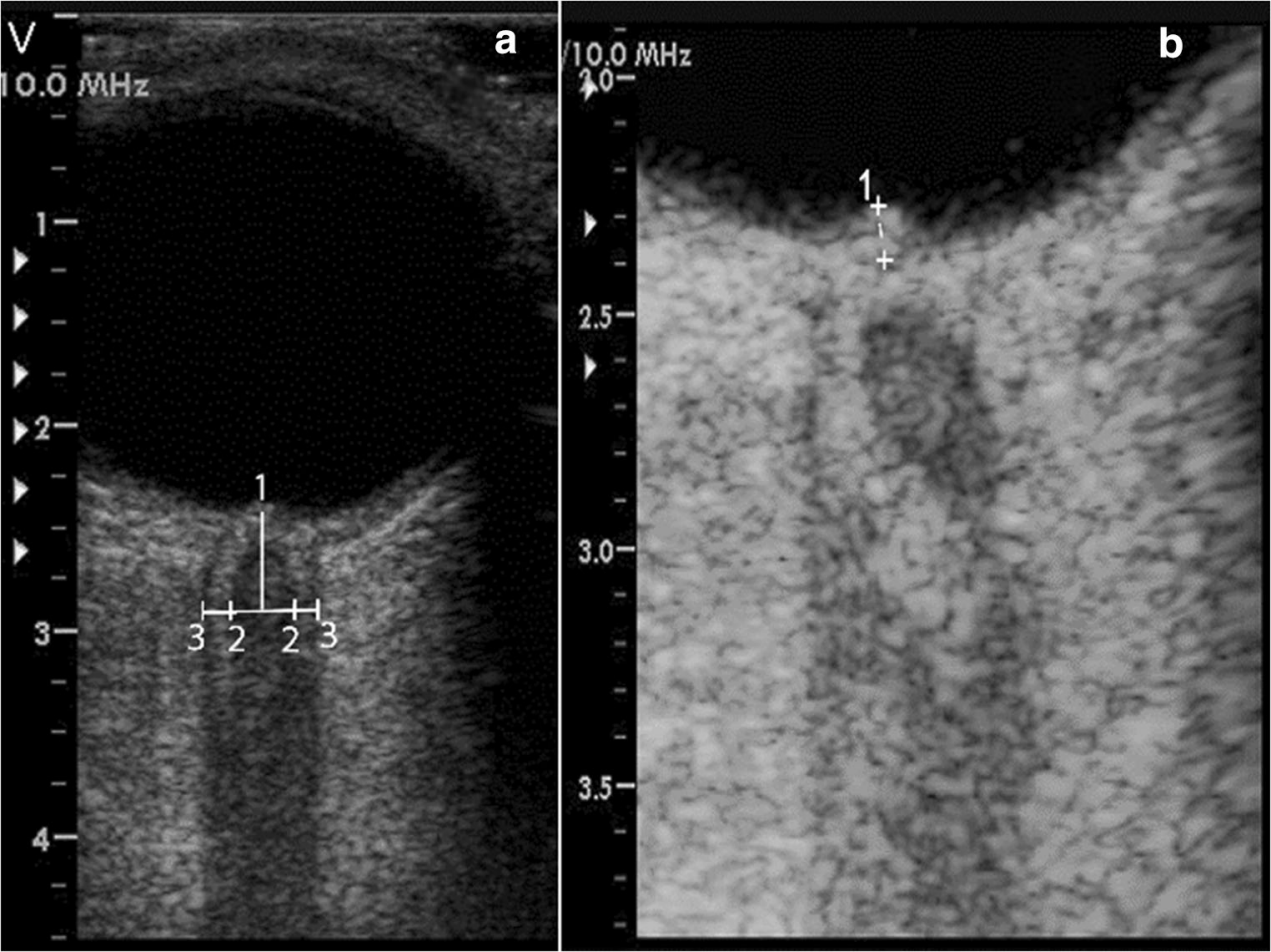
Transorbital sonography showing measurement of the ONSD (distance numbered as 3 while number 2 refers to the optic nerve diameter) performed 3mm behind the globe on the transverse plane (**a**). Three-times magnification of the optic disc in patient 2 demonstrating elevation of the papilla (**b**)

During the disease’s acute phase bilateral increases in ONSD were documented for all patients, while ODE was present just in two. Bilateral reduction of ONSD values was observed in three patients over time.

Lochner et al. showed the feasibility of TOS in supporting the diagnosis of PRES by detecting increased ICP and monitoring its development in a case report [4].

Both elevated ONSD and the presence of ODE are compatible with increased ICP as well as possible concomitant increased permeability and edema within the optic nerve in the acute stage of the disease. Of the two patients for whom it was measured, CSF pressure confirmed increased ICP thus confirming TOS findings. Compared with the ONSD dilation that occurs hyper-acutely, ODE may take days to develop [4]. Therefore, for the deceased patient, the short time between the onset of symptoms and TOS examination may explain why ODE was not present. However, in our experience, we highlight that only rarely the ONSD may not grow and sometimes ODE may also not be present so that ultrasonography findings may not be separated from clinical examination and course of the disease.

The normalization of TOS parameters over time corresponded to clinical improvement in the other patients, suggesting a valid monitoring role for this technique.

TOS is increasingly used as an alternative and non-invasive method for the detection of increased ICP. In fact, variation of ONSD follows ICP changes, allowing bedside monitoring of neurological conditions such as PRES in which multiple and strict evaluations are required [5]. Monitoring is also relevant outside the intensive care unit or similar settings where the invasive positioning of cerebral catheters for direct ICP measurements may not be possible. TOS fits perfectly in this context because of its great capacity to detect increased ICP (acute ONSD elevation and, if sufficiently extended, ODE) associated with the ability to re-normalize after ICP reduction. This versatility makes TOS particularly suitable also for verifying the effect of treatments and could encourage the reduction of furthermore expensive diagnostic tools like MR and exposure to radiation (CT), if clinical improvement matches favorable ultrasound parameters [6].

Diagnosing PRES is challenging and established diagnostic criteria do not exist. Clinical judgment is essential and must be coupled with serial brain MR or CT examinations, electroencephalography, and sometimes cerebral angiography. Lumbar puncture is usually performed to rule out other conditions (i.e., encephalitis) but may be also particularly useful to detect and quantify increased ICP. For early recognition of increased ICP in these patients, we encourage the prompt use of TOS as well as the measurement of CSF pressure, with the purpose to rapidly alert the intensive care units or consultants for potentially taking charge.

In conclusion, this study of a small series supports the application of TOS in patients with PRES for the identification of increased ICP and monitoring the disease’s development, but more evidence in larger samples is needed to further confirm our findings.

## Data Availability

Not applicable.
